# Matrons in Council: VIII. An All-Round View of Reconstruction

**Published:** 1917-09-29

**Authors:** 


### THE MATRONS' AND SISTERS' DEPARTMENT.

### MATRONS IN COUNCIL.

### VIII. An All-Round View of Reconstruction.

Reconstruction, so desirable and necessary in the nursing world, is not so entirely in the matron's hands as the able writer of an article entitled "Sparks from a Nurse's Anvil" would imply.

Probably all matrons would support Miss Nightingale's idea that nursing should be in the hands of educated women, but matrons have under existing conditions to train those who offer, and it speaks well for the efforts made both by the matrons of our training-schools and by the candidates who have not had great advantages of education that so many good nurses are produced, even while only a proportion of those who offer belong to the well-educated class.

The large training-school can and does secure a good proportion of those candidates who are better equipped for the nurse's calling, but, in order that throughout the nursing world our hospitals, infirmaries, schools, etc., should be staffed by women of education, it is necessary that the supply of candidates should be greatly augmented.

The writer of the article referred to is right in her view that more than examination results are necessary. By education one means far more than the ability to pass a technical examination, necessary as that is. It is the women of quick perception, trained to observe, before taking up nursing as a calling, who are so much needed.

Possibly a discussion amongst matrons might discover what steps could be taken to remove the causes that prevent so many possible candidates from taking the full training. Various causes are given—the long hours, the restrictions hospital work entails, the amount of time spent in "ward work," the limited time for study—but, above and beyond all, the rate of pay after training, when a woman is older than she would be on the completion of her training for any other calling.

Shorter hours and higher salaries, however, cannot become general until the *public* realise the necessity for them, and more generously support the hospitals. Matrons have long since realised the desirability of, and the necessity for, recreation and rest. Nurses forget that hospital training is to be obtained free of cost, and that in almost every case a salary is given during the time of training, whereas in other professions the training costs. This necessarily affects the after-rate of pay.

If being a matron does not mean a better nurse, it usually does and most certainly should demand wider experience. The aim of most matrons is to stand temporarily at least in the position of "mother" to her great family of patients and staff, and to give ungrudgingly her highest service in their interest.

It is indeed the wider experience, the broader outlook, the capacity for viewing from various standpoints that are the chief means whereby she may co-operate with those who work under her.

Elizabeth.

